# Unique Splicing of *Lrp5* in the Brain: A New Player in Neurodevelopment and Brain Maturation

**DOI:** 10.3390/ijms25126763

**Published:** 2024-06-20

**Authors:** Aureli Luquero, Noelia Pimentel, Gemma Vilahur, Lina Badimon, Maria Borrell-Pages

**Affiliations:** 1 Cardiovascular Program, Institut de Recerca de Sant Pau, 08025 Barcelona, Spain; aluquero@santpau.cat (A.L.); npimentel@santpau.cat (N.P.); gvilahur@santpau.cat (G.V.); lbadimon@santpau.cat (L.B.); 2 Biomedicine Doctorate Program, Universitat de Barcelona, 08007 Barcelona, Spain; 3 Centro Investigación Biomédica en Red-Cardiovascular (CIBER-CV), Instituto de Salud Carlos III, 28029 Madrid, Spain; 4 Universitat Autònoma de Barcelona, 08193 Barcelona, Spain

**Keywords:** *LRP5*, brain, RNA-seq, liver, transcriptome, synapse, retinoic acid

## Abstract

Low-density lipoprotein receptor-related protein 5 (LRP5) is a constitutively expressed receptor with observed roles in bone homeostasis, retinal development, and cardiac metabolism. However, the function of LRP5 in the brain remains unexplored. This study investigates LRP5’s role in the central nervous system by conducting an extensive analysis using RNA-seq tools and in silico assessments. Two protein-coding *Lrp5* transcripts are expressed in mice: full-length *Lrp5-201* and a truncated form encoded by *Lrp5-202*. *Wt* mice express *Lrp5-201* in the liver and brain and do not express the truncated form. *Lrp5*^−/−^ mice express *Lrp5-202* in the liver and brain and do not express *Lrp5-201* in the liver. Interestingly, *Lrp5*^−/−^ mouse brains show full-length *Lrp5-201* expression, suggesting that *LRP5* has a role in preserving brain function during development. Functional gene enrichment analysis on RNA-seq unveils dysregulated expression of genes associated with neuronal differentiation and synapse formation in the brains of *Lrp5*^−/−^ mice compared to *Wt* mice. Furthermore, Gene Set Enrichment Analysis highlights downregulated expression of genes involved in retinol and linoleic acid metabolism in *Lrp5*^−/−^ mouse brains. Tissue-specific alternative splicing of *Lrp5* in *Lrp5*^−/−^ mice supports that the expression of *LRP5* in the brain is needed for the correct synthesis of vitamins and fatty acids, and it is indispensable for correct brain development.

## 1. Introduction

Low-density lipoprotein receptor (LDLR)-related protein 5 (LRP5) induces the canonical WNT/β-catenin signalling pathway after the extracellular binding of WNT ligands or extracellular lipids [1,2,3]. LRP5 was identified when a loss-of-function mutation in *Arrow* (the *Drosophila melanogaster* homologue *LRP5* gene) generated flies without functional wings due to impaired development [4]. In normal conditions, the canonical WNT pathway is inactive, and there is constant phosphorylation, ubiquitination and degradation of β-catenin monomers [5,6]. Canonical WNT signalling activation through LRP5 leads to β-catenin stabilisation in the cytoplasm and translocation into the nucleus where it triggers the activation of the T cell factor/Lymphoid enhancer-binding factor 1 (TCF/LEF1) transcription factors [7,8]. TCF/LEF1 recruits other transcriptional co-activators to the promoter region of targeted genes such as *cyclin D1*, *Bmp2*, and *Opn*, inducing their expression [9,10].

Canonical WNT signalling is crucial in the central nervous system, as it regulates, amongst other processes, brain development, synapse formation, and neurogenesis [11,12,13,14,15,16]. Defects in canonical WNT signalling have been associated with central nervous system malfunction, including neural tube closure defects, medulloblastoma, bipolar disorder, schizophrenia, and Alzheimer’s disease [17,18,19]. In the brain, there is constitutive expression of LRP5 [20]. However, there is little knowledge on the role of LRP5 in brain development. In a human meta-analysis, two different single nucleotide polymorphisms (SNPs) in *LRP5* causing Ala1330Val amino acid changes have been associated with attention-deficit/hyperactivity disorder in females with altered brain maturation [21]. LRP5 is also necessary in zebrafish, where WNT3 binding to Frizzled1 activates the canonical WNT pathway that regulates brain development [22]. 

We have previously shown a role for LRP5 in extracranial tissues and organs. Indeed, LRP5 is involved in the healing process of the heart after myocardial infarctions in mice, pigs, and humans [23]. Furthermore, LRP5 expression is protective in the vascular wall, as LRP5 deficiency leads to increased aortic lipid accumulation, macrophage infiltration into the vessel wall, and increased pro-inflammatory cytokines in the blood of hypercholesterolemic mice [24,25]. Additionally, LRP5 is also involved in cholesterol ester accumulation in inflammatory cells [3], a process in which proprotein convertase subtilisin kexin 9 (PCSK9) is also involved [26]. Finally, LRP5 generates pro-survival signalling by stimulating the WNT/β-catenin pathway in neurons [27]. Taken together, these results indicate a protective and pro-survival role for LRP5 in tissue homeostasis.

*Lrp5*^−/−^ mice are generated by the insertion of an IRES-*LacZ*-*neomycin* cassette to interrupt the sixth exon of the mouse *Lrp5* gene at amino acid 373, generating a premature stop codon and blocking the synthesis of a full-length LRP5 protein [28]. This modification should affect all cells in mice. However, full-length LRP5 expression is observed in the brains of *Lrp5*^−/−^ mice. To understand these data, we analysed different organs of *Wt* and *Lrp5*^−/−^ mice.

## 2. Results

### 2.1. Non-Mendelian Pattern in Lrp5^−/−^ Mouse Births

**Figure 1:**
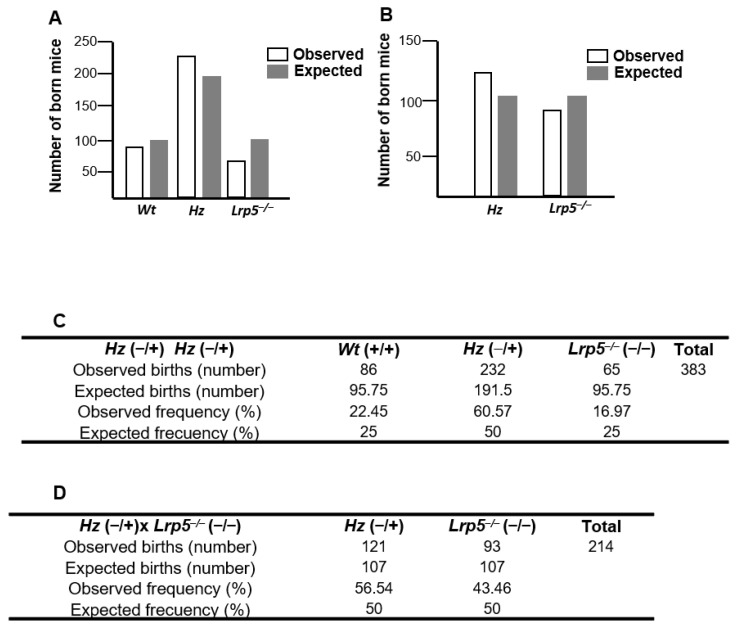
Analysis of *Lrp5*^−/−^ mouse offspring. Observed and expected births of wildtype (*Wt*; +/+), heterozygous (*Hz*; −/+), and knockout (*Lrp5*^−/−^; −/−) mice from (**A**,**C**) *Hz* crossbreeding (−/+ × −/+; *p* < 0.001) or (**B**,**D**) *Hz* and *Lrp5*^−/−^ crossbreeding (−/+ × −/−; *p* = 0.05) for over 10 years.

The analyses of the breeding of heterozygous (*Hz*; −/+ × −/+) mice from our *Lrp5*^−/−^ mouse colony showed that the offspring did not follow a Mendelian pattern. The observed births of *Lrp5*^−/−^ mice were less than expected (16.97% instead of the expected 25%), and there were increased *Hz* mouse births (60.57% instead of the expected 50%; Figure 1A,C). Similarly, the breeding of *Hz* mice to *Lrp5*^−/−^ mice (−/+ × −/−) also showed decreased births of *Lrp5*^−/−^ mice (93 births observed versus 107 expected; Figure 1B,D).

### 2.2. LRP5 Is Expressed in Brains of Lrp5^−/−^ Mice

**Figure 2:**
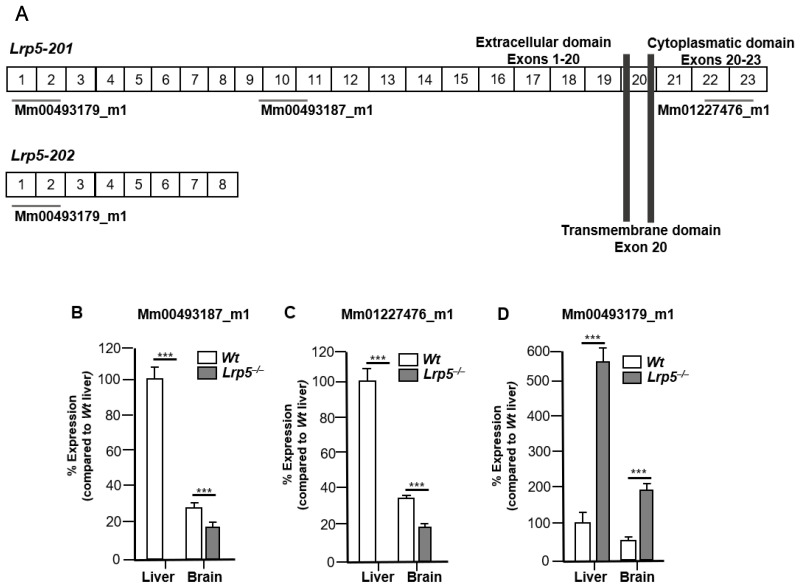
*LRP5* gene expression in the brains and livers of Wt and *Lrp5*^−/−^ mice. (**A**) The *Lrp5*-*201* transcript was detected by LP5 probes against exons 1–2, exons 9–10–11, and exons 22–23, whereas the *Lrp5*-*202* transcript was only detected by the *LRP5* probe against exons 1-2. *LRP5* gene expression in the liver and brain tissues of *Wt* and *Lrp5*^−/−^ mice using (**B**) *LRP5* probe Mm00493187_m1; (**C**) *LRP5* probe Mm_01227476; and (**D**) *LRP5* probe Mm_00493179. *** *p* < 0.001.

Two *Lrp5* protein-coding transcripts were generated from the *Lrp5 Mus musculus* gene by alternative splicing according to the Ensembl database [29]. The *Lrp5-201* transcript codes for the full-length *LRP5* protein, containing exons 1 to 23. The *Lrp5-202* transcript codes for a truncated protein containing exons 1 to 8; therefore, it codes for a short portion of the extracellular domain (Figure 2A). 

We first studied *Lrp5* gene expression in the brains and livers of *Wt* and *Lrp5*^−/−^ mice. Organs were analysed with the *LRP5* probe Mm_00493187, which detected exons 9–10–11. *LRP5* gene expression was expected in the livers and brains of *Wt* mice, and no *LRP5* gene expression was expected in the organs of *Lrp5*^−/−^ mice. Surprisingly, low but consistent expression of *LRP5* in the brains of *Lrp5*^−/−^ mice was detected (Figure 2B). To further confirm this unexpected result, we used a second probe, Mm_01227476, which detected exons 22–23. Again, *LRP5* expression was detected in the livers and brains of *Wt* mice and in the brains but not the livers of *Lrp5*^−/−^ mice (Figure 2C). We then tested a third probe, Mm_00493179, which detected exons 1–2–3 and therefore detected both the full-length *Lrp5-201* and the truncated *Lrp5-202* transcript. The expression of *LRP5* in the livers and brains of *Lrp5*^−/−^ mice was greater than the expression in *Wt* mice, indicating that the *Lrp5-202* transcript was expressed predominantly in the livers and brains of *Lrp5*^−/−^ mice (Figure 2D). These results indicate that *Lrp5* transcript expression is variable in different mouse tissues. 

### 2.3. Lrp5 Transcriptome Is Different in Livers and Brains of Lrp5^−/−^ Mice

**Figure 3:**
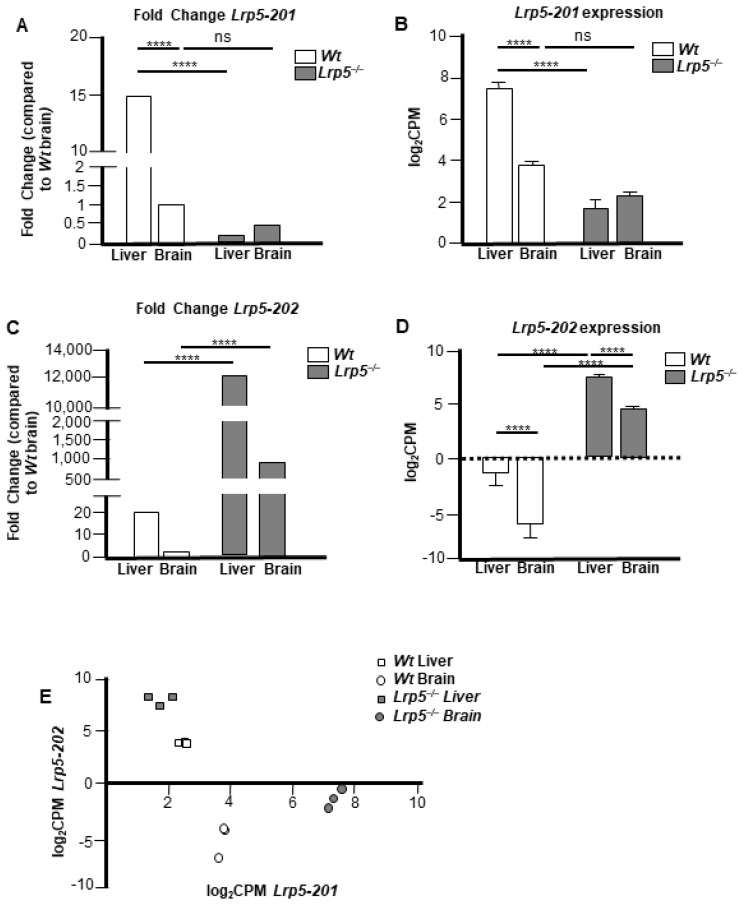
*Lrp5-201* and *Lrp5-202* transcript expression in the livers and brains of *Wt* and *Lrp5*^−/−^ mice. (**A**) Fold change in *Lrp5*-*201* transcript expression. (**B**) *Lrp5*-*201* transcript expression in the brains and livers of *Wt* and *Lrp5*^−/−^ mice expressed in log_2_CPM. (**C**) Same as (**A**) for *Lrp5*-*202*. (**D**) Same as (**B**) for *Lrp5*-*202*. (**E**) *Lrp5*-*201* transcript expression on the *X* axis and *Lrp5*-202 transcript expression on the *Y* axis for each tissue sample. Data are expressed as mean ± S.E.M. **** *p* < 0.0001; ns: non-statistically significant.

To further understand differential *Lrp5* gene expression in *Lrp5*^−/−^ mouse organs, samples of livers and brains were analysed by whole-tissue RNA-seq analyses. *Wt* mice livers showed 15-fold increased *Lrp5-201* expression compared to *Wt* mouse brain samples (Figure 3A), supporting the results from Figure 2B,C. Comparisons between *Wt* and *Lrp5*^−/−^ mouse liver samples revealed that *Wt* mice had an approximated 100-fold increase in *Lrp5-201* expression levels (Figure 3A,B). Contrarily, brain samples from *Wt* and *Lrp5*^−/−^ animals did not show statistically significant differences in *Lrp5-201* expression (Figure 3A,B).

*Lrp5-202* expression was increased in the livers (450-fold) and brains (850-fold) of *Lrp5*^−/−^ mice compared to *Wt* mice (Figure 3C,D). Similar to *Lrp5-201*, *Lrp5-202* transcript expression was higher in the livers than that in the brains of *Lrp5*^−/−^ mice (Figure 3D). These RNA-seq results confirm that the *Lrp5-201* transcript is expressed in the brains of *Lrp5*^−/−^ mice. More importantly, the RNA-seq analyses did not show statistical differences in *Lrp5-201* expression in *Wt* or *Lrp5*^−/−^ brain samples. The tissue expression of *Lrp5-201* and *Lrp5-202* using the log_2_CPM value in an *XY* axis indicated a similar *Lrp5* transcript pattern expression for each sample of the same group (Figure 3E).

### 2.4. LRP5 Deficiency Leads to Alterations in the Transcriptome of Livers and Brains

**Figure 4:**
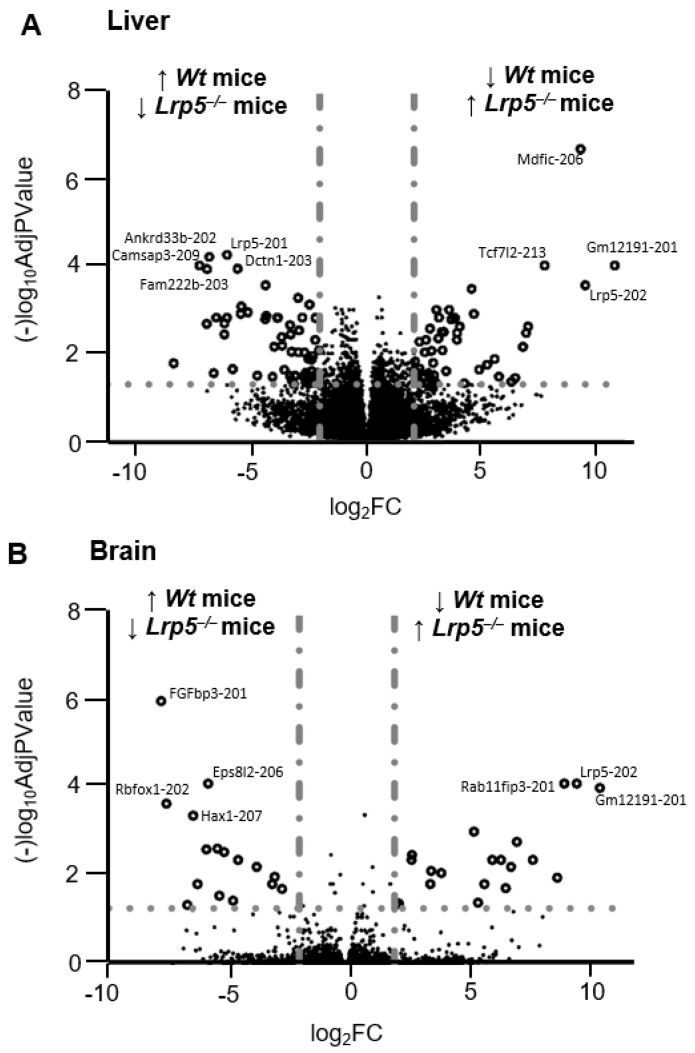
Volcano plots for liver and brain samples. Volcano plot comparing transcript expression in (**A**) livers of *Lrp5*^−/−^ mice vs. livers of Wt mice and in (**B**) brains of *Lrp5*^−/−^ mice vs. brains of *Wt* mice. Data are expressed as log_2_FC on the *X* axis and as (−)log_10_AdjPvalue on the *Y* axis. Transcripts above the horizontal grey dotted line (···) show significantly modified expression in *Lrp5*^−/−^ mice compared to *Wt* mice. Vertical grey bar-dot lines (― · ― ·) indicate thresholds where transcripts reduced expression by ½-fold or increased by 2-fold in mouse *Lrp5*^−/−^ tissue compared to *Wt* mice tissue. Empty dots (○) indicate transcripts with highly modified expression in *Lrp5*^−/−^ tissues. ↑ indicates that the transcript expression is significantly higher in animals of the genotype and ↓ indicates that transcript expression is significantly lower in animals of the genotype.

To assess if *LRP5* deficiency can modulate the expression of other genes, we compared gene expression in the livers of *Wt* and *Lrp5*^−/−^ mice. The transcription factor encoded in transcript *Mdfic-206*, with other transcripts including non-protein coding *Tcf2l7-213* or *Gm12191-201* and the *LRP5* truncated isoform *Lrp5-202*, were significantly reduced in the livers of *Wt* mice compared to the livers of *Lrp5*^−/−^ mice, indicating that *Lrp5-201* deficiency modifies the liver transcriptomic pattern (Figure 4A). Table 1 shows a list of the transcripts that were significantly modified in the livers of *Lrp5*^−/−^ mice compared to *Wt* mice. When the brain samples of *Wt* and *Lrp5*^−/−^ mice were analysed, the results showed increased expression of *Lrp5-202* transcripts in the brains of *Lrp5*^−/−^ animals. Other transcripts with modified expression in *Lrp5*^−/−^ mouse brains compared to *Wt* mouse brains included protein-coding transcripts *Rab11fip3-201*, *FGFbp3-201*, or *Rbfox1-202* (Figure 4B). Table 2 shows a list of the transcripts that were significantly modified in the brains of *Lrp5*^−/−^ mice compared to *Wt* mice.

### 2.5. Lrp5 Quantity Is Different in Livers and Brains of Lrp5^−/−^ Mice

**Figure 5:**
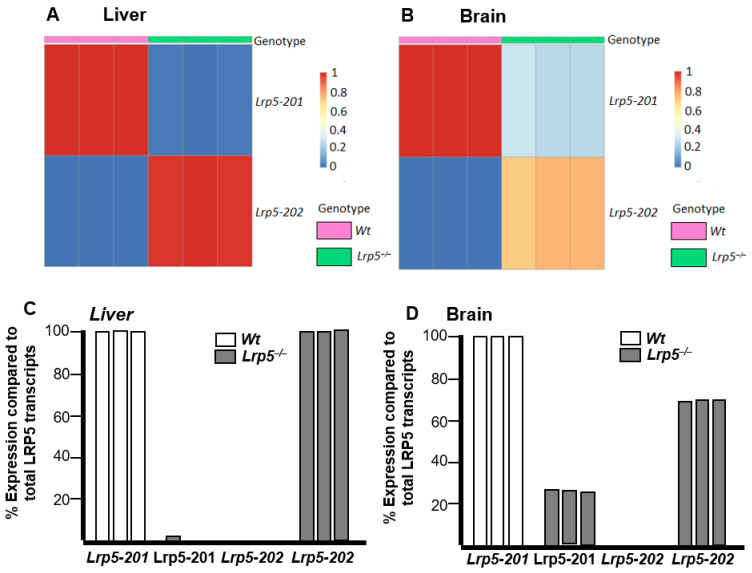
*Lrp5* transcript variability depending on tissue and mouse genotype. Heat map with the number of *Lrp5-201* and *Lrp5-202* transcripts in the (**A**) livers and (**B**) brains of *Wt* and *Lrp5*^−/−^ mice. *Lrp5-201* and *Lrp5-202* expression compared to total *Lrp5* transcripts in *Wt* and *Lrp5*^−/−^ mouse (**C**) livers and (**D**) brains.

The balance of the different *Lrp5* transcripts in each tissue was then evaluated. Differential transcript usage (DTU) analysis showed that the livers and brains of *Wt* mice expressed only the *Lrp5-201* transcript (Figure 5A,B). In *Lrp5*^−/−^ mice, the liver’s *Lrp5-201* transcript accounted for less than 2% of *Lrp5* transcripts, whereas *Lrp5-202* accounted for more than 98% (Figure 5C). However, in the brains of *Lrp5*^−/−^ mice, *Lrp5-201* accounted for 27% of *Lrp5*-encoding transcripts, whereas 73% were *Lrp5-202* transcripts (Figure 5D). 

### 2.6. Functional Studies Show Modified Functions in Brains of Lrp5^−/−^ Mice

**Figure 6:**
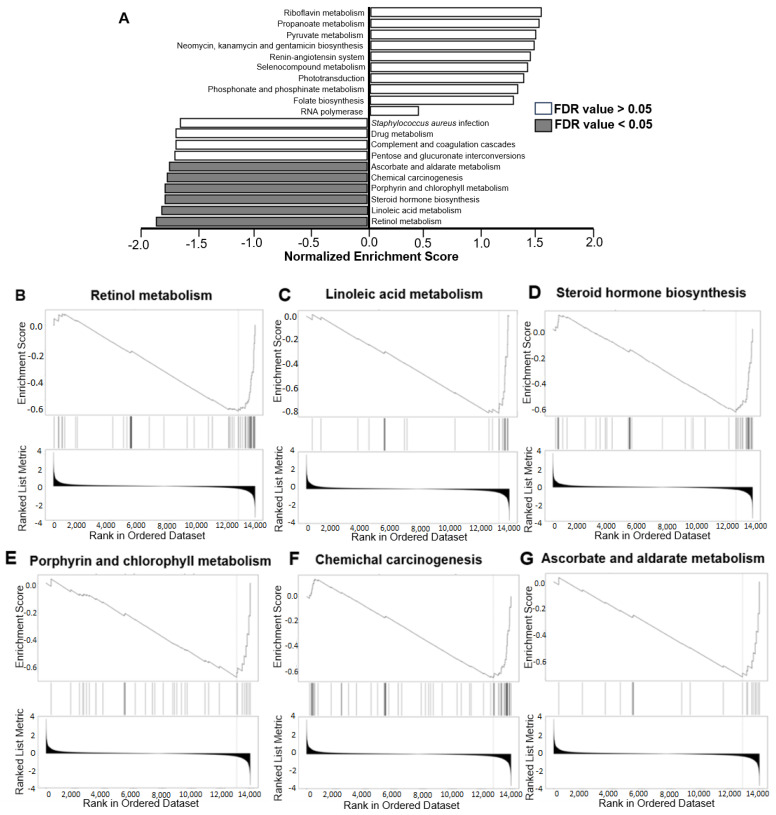
Gene Set Enrichment Analyses (GSEA) on the brains of Wt and Lrp5^−/−^ mice. (**A**) List of the top 10 most dysregulated pathways in the brains of *Lrp5*^−/−^ mice. Positive values on the *X* axis indicate upregulation, and negative values on the *X* axis indicate downregulation compared to the brains of *Wt* mice. (**B**–**G**) GSEA plots for pathways with FDR < 0.05, (**B**) retinol metabolism, (**C**) linoelic acid metabolism, (**D**) steroid hormone biosynthesis, (**E**) porphyrin and chlorophyll metabolism, (**F**) chemichal carcinogenesis, and (**G**) ascorbate and aldarate metabolism. All gene sets available in the Gene Ontology database were considered. Figures (**B**–**G**): *X*-axis is the Rank in Ordered Dataset ranging from 0 to 14,000; superior *Y*-axis is the Enrichment Score ranging from 0.0 to −0.8; inferior *Y*-axis is the Ranked List Metric ranging from 4 to −4.

To study the effects of *LRP5* deficiency on brain functionality, functional gene enrichment analysis was performed on RNA-seq data from the brains of *Wt* and *Lrp5*^−/−^ mice, showing that *LRP5* transcripts are associated with specific functions of the brain, including “Cell morphogenesis involved in neuron differentiation” and “Synapsis formation” (Table 3). Gene Set Enrichment Analysis (GSEA) showed that genes involved in retinol and linoleic acid metabolism are downregulated in the brains of *Lrp5*^−/−^ mice compared to *Wt* mice (Figure 6A–C). Other pathways with downregulated gene expression in *Lrp5*^−/−^ mouse brains are steroid hormone biosynthesis, porphyrin and chlorophyll metabolism, chemical carcinogenesis, and ascorbate and aldarate metabolism (Figure 6D–G). 

**Figure 7:**
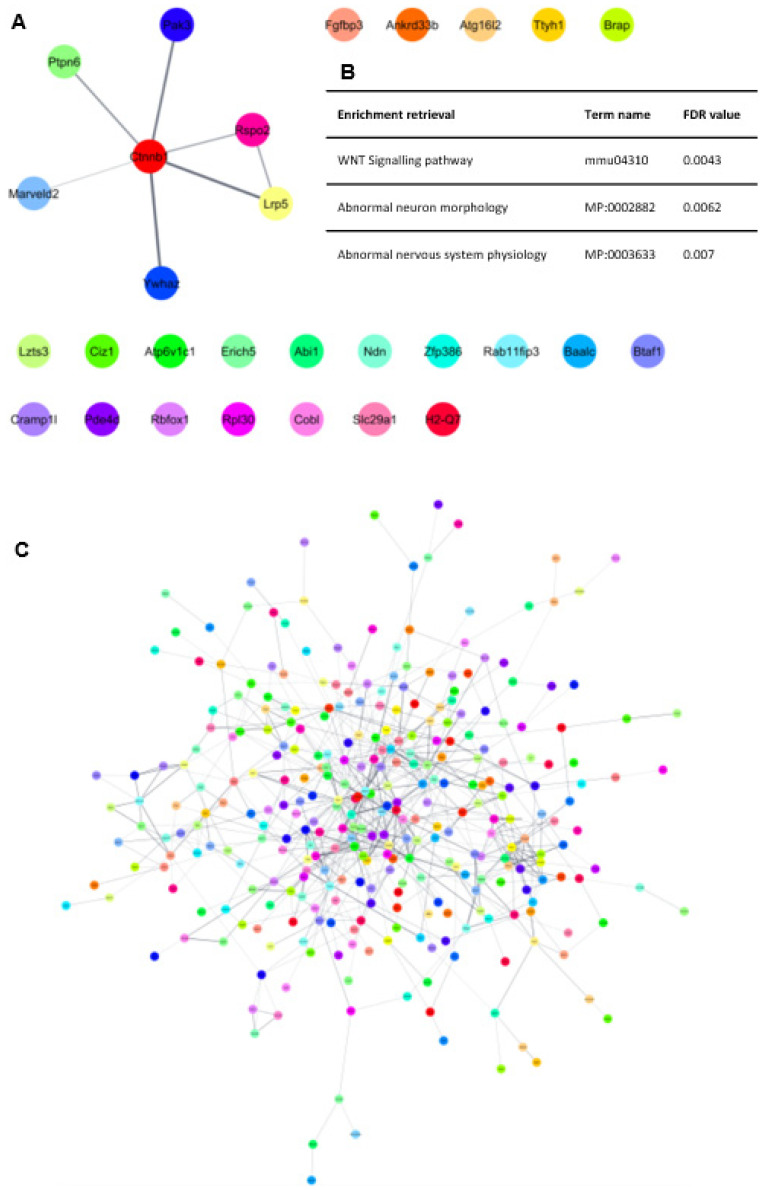
Network analysis of RNA-seq data. (**A**) Protein–protein interaction network of transcripts with modified expression in Lrp5^−/−^ mice brains. Only interactions with a confidence score higher than 0.4 are shown. A β-catenin node was added to generate a cluster of interacting proteins. Singletons were included in the figure to show that the majority of proteins with altered expression did not interact with each other. (**B**) Table showing functional gene enrichment retrieved from proteins forming the cluster in A. Singletons were not included for the enrichment. Term names and FDR data are included in the table. (**C**) Protein–protein interaction network of transcripts with modified expression in *Lrp5*^−/−^ mice livers. Only interactions with a confidence score higher than 0.4 are shown.

Network analysis using Cytoscape software based on the STRING database showed that several genes with modified expression in *Lrp5*^−/−^ mice not only participate in the WNT/β-catenin signalling pathway but are also involved in abnormal neuron morphology and abnormal central nervous system physiology (Figure 7A,B). All these findings suggest that dysregulation in the WNT/β-catenin pathway can be the cause for a deficient retinol acid and linoleic acid metabolism, which, in turn, can produce deficits in neuron differentiation and neuron synapsis formation.

### 2.7. Functional Studies Show Impaired Functions in Livers of Lrp5^−/−^ Mice

Functional gene enrichment analysis on RNA-seq data from the livers of *Wt* and *Lrp5*^−/−^ mice showed that over 300 liver functions were significantly modified in *Lrp5*^−/−^ mice compared to *Wt* mice, including processes involving cellular and metabolic pathways (Table 4). Liver RNA-seq data were also subjected to network analysis, resulting in 319 proteins that had their expression modified in the livers of *Lrp5*^−/−^ mice (Figure 7C). Furthermore, clustering of the network followed by functional gene enrichment analysis revealed that each group of closely interacting proteins are associated with specific modified functions (Supplementary Figure S1, Supplementary Table S1). Network analyses support that the livers of *Lrp5*^−/−^ mice were more severely affected than their brains by the loss of *Lrp5-201* expression as more functions were altered in their gene expression profiles.

## 3. Discussion

We analysed the breeding of our *Lrp5*^−/−^ mice colony in the last 10 years and observed that, after mating heterozygous mice, *Lrp5*^−/−^ mice were born less frequently than expected. Furthermore, the mating of heterozygous with knockout mice also showed reduced births of *Lrp5*^−/−^ mice. This finding suggests that *LRP5* expression might be essential for mouse embryonic development. 

*Lrp5-201* is not expressed in the peripheral tissues of *Lrp5*^−/−^ mice, including the liver, aorta, heart, spleen, and jejunum [27], but it is expressed in their brains, showing a mosaic expression of the *Lrp5-201* transcript in *Lrp5*^−/−^ mice. Indeed, the protein expression pattern of full-length *LRP5* resembles that of gene *Lrp5-201.* Interestingly, all *Lrp5*^−/−^ mice showed similar *Lrp5-201* expression in their brains, supporting a role for *Lrp5-201* in survival. *Lrp5*^−/−^ mice expressed significantly fewer *Lrp5-201* transcripts than *Wt* mice in the brain. The insertion of the IRES-*LacZ-Neomycin* cassette at the end of exon 6 abrogated full-length *LRP5* transcript formation; however, the brain splicing machinery could avoid the inserted sequence producing the *Lrp5-201* transcript. The inserted cassette probably hampered the efficiency of the splicing process, as the immature *Lrp5* transcript was mostly converted into an *Lrp5-202* transcript. 

Because *LRP5* was not expressed in extracranial tissues in *Lrp5*^−/−^ mice, *LRP5* must not be required in the organogenesis of extracranial organs. However, *LRP5* is active after hypercholesterolemia or ischemia [3,26,30,31], indicating that particular RNA splicing in the *Lrp5* transcript must occur exclusively in the brains of *Lrp5*^−/−^ mice to generate an *Lrp5* transcript similar to full-length *Lrp5-201* that can generate a functional protein.

*Lrp5-202* expression in the livers of *Lrp5*^−/−^ mice was higher than that of *Lrp5-201* in the livers of *Wt* mice. This indicates that a lack of *Lrp5-201* induces the synthesis of high levels of *Lrp5-202* truncated transcripts in an attempt, probably, to counterbalance the loss of *LRP5* function. 

Similarly, reduced expression of *Lrp5-201* transcripts in the brains of *Lrp5*^−/−^ mice led to the overexpression of *Lrp5-202*. This could be explained because of an insufficient quantity of full-length *LRP5* proteins being produced by the *Lrp5-201* transcript or that the full-length *LRP5* protein encoded by the *Lrp5-201* transcript could not reproduce *LRP5*’s normal functions. We hypothesise that only those embryos that showed brain *Lrp5-201* transcript expression were viable. We showed that *Lrp5*^−/−^ mice had similar brain expression of *Lrp5-201* transcripts (Figure 5B), further supporting that mouse embryos that do not express more than 25% of *Lrp5-201* transcripts are not viable and probably die during the early gestation stages.

RNA-seq analysis revealed differential expression of *Lrp5-201* and *Lrp5-202* transcripts in the livers and brains of *Wt* mice compared to their *Lrp5*^−/−^ littermates. *Lrp5*^−/−^ mouse brains showed modified expression of 48 mature RNAs, 35 of which were protein coding mRNAs. In contrast, *Lrp5*^−/−^ mouse livers showed modified expression of 546 transcripts, 488 of them being protein-coding mRNAs. This finding suggests that, by the preservation of full-length *LRP5* expression, the brain transcriptome is less modified than the liver transcriptome, which shows a complete loss of *LRP5* expression and function. This finding is further confirmed by the network in silico analysis, in which brain altered transcripts needed at least the β-catenin node addition to generate a minimum network of interacting proteins. Hence, this finding supports our hypothesis that, in *Lrp5*^−/−^ mice, there is expression of fully active *LRP5* and that the *LRP5* brain’s expression must be preserved to ensure survival. Of note, we believe that the generation of *Lrp5-201* transcripts in *Lrp5*^−/−^ mouse brains is not an efficient process, as most of the *LRP5* transcripts synthesised were *Lrp5-202* transcripts. Hence, in order to have enough functional *LRP5* in the brains of *Lrp5*^−/−^ mice, vast quantities of *Lrp5-202* transcripts were synthesised as a by-product.

Liver altered transcripts generated a huge network with hundreds of interacting proteins. Further clustering of liver genes followed by functional gene enrichment analysis showed that multiple functions were dysregulated in the livers of *Lrp5*^−/−^. These functions comprise essential cellular metabolic pathways, including regulation of transcription, control of mRNA splicing, catabolism, autophagy, and others. 

Functional gene enrichment analysis in *Wt* and *Lrp5*^−/−^ mouse brains revealed that different genes are involved in the same cellular functions. Also, the proteins can be grouped and associated with different pathways, including neuronal differentiation and synapsis formation. Therefore, downregulation of these pathways could explain the low number of *Lrp5*^−/−^ mouse births. Furthermore, if full-length *Lrp5-201* expression was completely abolished from *Lrp5*^−/−^ mouse brains, increased modified gene transcripts (similar to the liver samples) would be expected. 

GSEA revealed significant downregulation of genes associated with retinol, linoleic acid, and other biosynthetic pathways in the brains of *Lrp5*^−/−^ mice. A deficit in retinol acid metabolism is associated with impaired neuronal plasticity and defects in the development of the central nervous system, as retinoic acid has very specific effects on neuronal differentiation [32,33,34,35]. Linoleic acid and derivates have also been involved in mouse reflex maturation and memory improvement [36], and elevated linoleic acid concentrations in the blood can lead to mouse brain malfunction and inflammation [37]. Our findings show downregulation of the retinol and linoleic acid pathways in the brains of *Lrp5*^−/−^ mice, suggesting that a reduction in the expression of full-length *LRP5* causes deficits in neuronal differentiation and synapsis formation.

Full-length *LRP5* is transported to the cell membrane in endosomal bodies from the endoplasmic reticulum [38]. *LRP5*’s transmembrane domain allows the receptor’s insertion into the plasma membrane. An artificial dominant-negative soluble form of *LRP5* lacking the transmembrane and cytoplasmatic domains has been used as a WNT/β-catenin pathway inhibitor. Soluble *LRP5* contains the full extracellular protein sequence (exons 1–19) and shows *LRP5* antagonist properties preventing WNT ligands from binding full-length *LRP5*, suppressing the expression of tumorigenic and metastatic proteins and inducing an epithelial to mesenchymal transition in Saos-2 cells [39]. Soluble *LRP5* also reduces 143B cell tumour growth in nude mice [40]. The *Lrp5-202* transcript encodes for a protein containing only a fraction of the extracellular domain (exons 1–6), opening the possibility that it can also act as a WNT pathway repressor; however, functional studies are needed to determine the possible roles for this isoform. To the best of our knowledge, no protein similar to that encoded by the *Lrp5-202* transcript has been described. 

This study highlights the importance of *LRP5* expression in the brain. We observed fewer births of mice with a *Lrp5*^−/−^ genotype as opposed to a *Wt* genotype and were able to demonstrate that mice unable to express full-length *LRP5* in the brain die during embryonic stages. Furthermore, we showed a protective mechanism that involves the alternative splicing of *Lrp5* transcripts to avoid a premature stop codon and generate a full-length *Lrp5* transcript in mouse brains, suggesting a role for *LRP5* in the preservation of brain function during development. Finally, Gene Set Enrichment Analysis highlighted the downregulated expression of genes involved in retinol and linoleic acid metabolism in *Lrp5*^−/−^ mouse brains, supporting that the expression of *LRP5* in the brain is needed for the correct synthesis of vitamins and fatty acids, and it indispensable for correct brain development.

## 4. Materials and Methods

### 4.1. Animal Models and Experimental Design

Genes and proteins from mouse and human samples are written in accordance with the guidelines from the ‘‘International Committee on Standardized Genetic Nomenclature for Mice and the Rat Genome’’, 2010. Briefly, mouse genes and transcripts are written in italics (*Lrp5*), human genes are written in italics and capital block letters (*LRP5*) and proteins from the two species are written in straight capital block letters (*LRP5*) [41].

The study protocols for mice were approved by the institutional Animal Care and Use Committee (ICCC051/5422) and authorised by the local government commission. Animal procedures conformed to guidelines published in directive 2010/63/EU of the European Parliament and the “Position of the American Heart Association on Research Animal use” (11 November 1984). At the research institute, we are committed to the “3R”s principle, using the minimum number of animals required to accomplish statistical significance. 

*Lrp5*^−/−^ mice were a kind gift from Dr. Bart Williams [42]. Mouse strains were maintained in a C57bl/6J genetic background. Animals were housed in cages under controlled monitoring of temperature (21 ± 2 °C) on a 12 h light/dark cycle with food and water ad libitum. Genotyping was performed on mice 4 weeks after birth using PCR amplification from DNA extracted from tail biopsies, resulting in the identification of *Wt*, *Lrp5^−/+^*, or *Lrp5*^−/−^ mouse genotypes. Heterozygous *Lrp5^−/+^* mice were discarded for this work. Adult animals were sacrificed at 16–18 weeks old after terminal anaesthesia (ketamine/medetomidine, 75 mg/kg and 1 mg/kg, respectively, i.p.). Mouse organs were collected, washed extensively in sterile saline, and frozen immediately in liquid nitrogen.

### 4.2. RNA Isolation and Real-Time PCR

Frozen mouse tissue samples from livers and brains were smashed to dust using mortar and liquid nitrogen. Pulverised tissues were processed for RNA extraction using RNEasy Kit from Qiagen (Qiagen, Hilden, Germany). Total RNA concentration and purity were determined using a Nanodrop ND-1000 Spectrophotometer (Nanodrop Technologies, Inc., Wilmington, DE, USA). For purity standards, only samples in which A260/A280 ratios were between 1.8 and 2.1 were considered acceptable. cDNA synthesis was performed using 1 µg RNA and cDNA reverse-transcription kit (Applied Biosystems, Foster City, CA, USA). The generated cDNA was amplified by real-time polymerase chain reaction in a 7900HT Fast Real-Time PCR System (Applied Biosystems, Foster City, CA, USA) using probes from Applied Biosystems. Different *LRP5* probes were used to detect different regions of the transcript: for exons 1–2, probe Mm00493179_m1 was used; for exons 9–10–11, probe Mm00493187_m1 was used; and for exons 22–23, probe Mm01227476_m1 was used (ThermoFisher, Waltham, MA, USA). Results were normalised against r18s mRNA expression, which was measured using a specific r18s probe from Applied Biosystems.

### 4.3. RNA-Seq Analysis

RNA was isolated from *Wt* or *Lrp5*^−/−^ mouse brain and liver samples using the RNAEasy extraction kit from Qiagen. RNA samples were sent to “Centro Nacional de Análisis Genómico” (CNAG) for RNA sequencing and analysis. RNA purity was checked by A260/A280 and A260/A230 ratios, and only RNA with ratios between 1.8 and 2.1 was used for this analysis. RNA integrity was further analysed by Bioanalyzer 2100 (Agilent Tech, Santa Clara, CA, USA) using an Agilent RNA nano 6000 kit (Agilent Tech, Santa Clara, CA, USA), and only RNAs with an RNA Integrity Number >8 were accepted. RNA-seq reads were trimmed with TrimGalore (version 0.6.10, 2 Feb 2023) [43] and mapped against the *Mus musculus* reference genome (GRCm39) with STAR/2.7.8a [44] using ENCODE parameters. Genes and isoforms were quantified with RSEM/1.3.0 [45] with default parameters using the gencode.M32 annotation. Differential expression was performed with the R Package limma-voom (https://bioconductor.org/packages/release/bioc/html/limma.html (accessed on 15 May 2024)) [46], and differential transcript usage was determined with the DTUrtle R Package (https://tobitekath.github.io/DTUrtle/ (accessed on 15 May 2024)) [47].

### 4.4. In Silico Systems Biology Analysis

Data from the RNA-seq analysis of differentially expressed genes were imported into Cytoscape 3.10.0 to build a protein–protein interaction (PPI) network based on STRING database interaction data. The confidence cut-off value was set to 0.4. An additional node was added to the brain network to generate a minimal network of interacting proteins. To generate the networks, only protein-coding transcripts that showed altered expression between tissues from animals of different genotypes in the RNA-seq analysis were included for this study. In order to identify protein–protein interaction clusters, the community cluster strategy GLay algorithm was used. Functional enrichment was performed with g:profiler [48] using as input a list of differentially expressed genes.

Gene Set Enrichment Analysis (GSEA) was performed using WebGestalt: update 2013 (Web-based Gene Set Analysis Toolkit) [49], and the “Geneontology” functional database was selected for the analysis. The top 10 most significant categories are shown in the results. Significance was considered for FDR values < 0.05. For GSEA, we used log_2_FC values, comparing the transcript expression of *Lrp5*^−/−^ brain samples against *Wt* brain samples to rank genes. 

### 4.5. Statistical Analysis

Experimental data were expressed as mean ± S.E.M. To assess alterations in the frequency of the genotypes of the different born mice, the chi-squared goodness-of-fit test was used. To establish significance, data were subjected to a one-way ANOVA followed by Bonferroni’s multiple-comparisons test using GraphPad Prism software statistical package 10 (GraphPad Software, San Diego, CA, USA). The criterion for significance was set as a *p* value ≤ 0.05.

## 5. Conclusions

We describe for the first time that *LRP5* pre-mRNA undergoes differential splicing during mRNA maturation and that this splicing is tissue-dependent. *Lrp5*^−/−^ mice that are unable to generate brain full-length *LRP5* cannot develop during the embryonic stages, explaining the unbalanced Mendelian pattern observed at birth. Our results support that *LRP5*’s brain expression is needed for the correct synthesis of vitamins and fatty acids, and subsequently, it is indispensable for normal brain development.

## Figures and Tables

**Table 1:** List of transcripts with significantly altered expression in livers of *Lrp5*^−/−^ mice compared to *Wt* mice. *p* value < 0.05.

Gene Transcripts with Altered Expression in Livers of *Lrp5*^−/−^ Mice								
*Mdfic-206*	*Myo5a-204*	*Lpin2-204*	*Wnk2-211*	*Rida-201*	*Fbxo16-204*	*Otud1-201*	*Ranbp10-201*	*1500011B03Rik-204*
*Lrp5-201*	*Dpys-201*	*Gabrb3-201*	*Tlcd4-207*	*Xlr3a-201*	*Irf6-201*	*Aplp2-203*	*Atp5pb-203*	*0610030E20Rik-201*
*Fam222b-203*	*Slc13a3-201*	*Nat8f2-201*	*Wdr77-201*	*Eml1-202*	*Kif26b-202*	*Tbp-211*	*Med13l-201*	*1110032F04Rik-201*
*Tcf7l2-213*	*Ppm1k-201*	*Papola-202*	*Serpinc1-207*	*Dph7-201*	*Zhx3-202*	*Ifnar2-201*	*Fech-201*	*D5Ertd579e-201*
*Camsap3-209*	*Bend6-201*	*Sptan1-202*	*Fus-204*	*Fzd8-201*	*Zfp703-202*	*Gmppb-202*	*Tmem25-204*	*AW209491-202*
*Dctn1-203*	*Fgd6-201*	*Hnrnpa1-202*	*Gsap-201*	*Lipa-201*	*Yy1-201*	*Mat1a-201*	*Btg1-202*	*Cdc42bpb-201*
*Ankrd33b-202*	*Clk3-201*	*Zmynd8-203*	*Xpo4-209*	*Ppm1b-201*	*Eif5-201*	*Lrrc73-204*	*Bptf-203*	*2810021J22Rik-201*
*Lrp5-202*	*Hddc3-208*	*Dpys-202*	*mt-Atp6-201*	*St6gal1-205*	*Aacs-201*	*Ide-201*	*Ankrd11-202*	*A630089N07Rik-202*
*Ociad2-205*	*Stom-201*	*Pxmp2-201*	*Tab2-204*	*Crebrf-201*	*Relch-205*	*Tmpo-201*	*Mcfd2-204*	*2410002F23Rik-202*
*Meis3-205*	*Tspyl5-201*	*Slc8b1-202*	*Cyp39a1-203*	*Cog8-201*	*Map2k3-201*	*N4bp2l2-201*	*Pwwp2a-203*	*Nr1i2-201*
*Cps1-201*	*Zfand5-205*	*Irgm1-202*	*Bet1l-201*	*Rhod-201*	*Hes6-202*	*Nr1h2-201*	*Dcaf12l1-202*	*Wbp1l-201*
*Slc15a2-205*	*Inpp5f-208*	*Myef2-201*	*Rab9-202*	*Zfp120-201*	*Wnk2-201*	*Dst-201*	*Elf1-201*	*Srp54a-202*
*Rapgef1-207*	*Jmy-201*	*Rsph1-201*	*Pxmp4-201*	*Znfx1-201*	*Tfdp1-204*	*Gpbp1-202*	*Mphosph8-201*	*Slc4a2-201*
*Pnrc1-201*	*Traf4-201*	*Dclk2-206*	*Rnf186-201*	*Csnk1d-202*	*Scaf11-204*	*Rabep1-207*	*Chd4-203*	*Dtymk-201*
*Gamt-202*	*Brap-201*	*Srsf7-201*	*Kdm2a-202*	*Etv6-202*	*Pik3r1-202*	*Bhlhe41-201*	*Pi4ka-201*	*Ip6k2-206*
*Prxl2c-207*	*Gria4-203*	*Pdcd11-201*	*Esyt2-201*	*Stxbp3-201*	*Ahctf1-201*	*Slc38a2-201*	*Pkp4-211*	*Gemin5-205*
*Aktip-204*	*Gabarapl1-201*	*Slc39a14-202*	*Qdpr-201*	*Sgsh-201*	*Atp6ap2-201*	*Gpbp1l1-201*	*Per2-201*	*Azin1-203*
*Zfyve1-201*	*Irs2-201*	*Elfn2-201*	*Dnajc13-203*	*Rsph4a-201*	*Rai1-202*	*Cmtm4-201*	*Slc43a1-201*	*Zrsr2-201*
*Slc25a33-201*	*Spns2-201*	*Papss2-201*	*Mxd4-201*	*Fah-201*	*Epm2aip1-201*	*Cyp2c70-201*	*Grb10-203*	*Ttc14-211*
*Tpm1-215*	*Dtx3l-201*	*Uox-201*	*Hdac5-202*	*Csad-211*	*Irf2bp2-201*	*Pfkfb2-204*	*Dusp3-201*	*Snrnp48-201*
*Klf11-201*	*Maoa-201*	*Inf2-201*	*Pspc1-201*	*Prpsap1-201*	*Tbc1d20-201*	*Flcn-203*	*Cebpb-201*	*Gpcpd1-202*
*Dctn1-202*	*Tor1aip2-205*	*Epc2-201*	*Rabggtb-201*	*Abat-201*	*Bdp1-204*	*Hbp1-202*	*Atrip-201*	*Iigp1-202*
*Evi5l-207*	*Pparg-202*	*Dennd11-202*	*Fn3krp-201*	*Serpinf2-202*	*Jmjd1c-206*	*C9orf72-203*	*Riok2-201*	*Sstr4-201*
*Klhl24-201*	*Cnppd1-201*	*Foxp4-208*	*Cdc42bpg-201*	*Hnrnpa3-203*	*Fnip1-201*	*Smc5-202*	*Bzw1-201*	*Unc13b-201*
*Cyria-205*	*Dach1-202*	*Stk24-201*	*Aldh3a2-202*	*Tro-204*	*Tmf1-202*	*Atxn2-201*	*Slc25a47-201*	*Ciart-201*
*Il13ra1-201*	*Erbb3-201*	*Serpinb9-201*	*Agxt2-204*	*Ubiad1-201*	*Clic5-203*	*Chn2-202*	*Zfp955a-201*	*Elac1-201*
*Trim46-201*	*Spryd4-201*	*Ilrun-203*	*Pcdh1-204*	*Gorasp1-201*	*Upp2-202*	*Atpsckmt-201*	*Ap4m1-201*	*Nfyc-204*
*Gclc-201*	*Sesn2-201*	*Mmab-201*	*Zfand6-208*	*Dyrk3-201*	*Csnk1g1-202*	*Stat5b-201*	*Hsd17b7-201*	*Ipmk-203*
*Dgkb-203*	*Dlg4-205*	*Thrsp-201*	*Fbxl19-201*	*Blvrb-201*	*Slc25a22-225*	*Lats2-201*	*Fbxl3-201*	*Mef2d-204*
*Gla-201*	*Nr2c2-201*	*Klhl42-201*	*Ppp1r3b-201*	*Pcsk9-201*	*Paqr5-201*	*Sf3a1-201*	*Nars-205*	*Rnf125-202*
*Muc3a-202*	*Stard4-201*	*Tstd3-201*	*Inf2-203*	*Cpeb2-202*	*Tmc6-201*	*Map3k11-201*	*Stau2-212*	*Dcaf11-202*
*Aldh1l1-201*	*Slc38a3-209*	*Bcan-201*	*Mtdh-202*	*Gnpnat1-201*	*Rnd1-201*	*Tfe3-201*	*Ss18l2-201*	*Dhtkd1-202*
*Smurf1-203*	*Ccng2-201*	*Atad3a-201*	*Mink1-201*	*Zfp266-202*	*Arhgef3-202*	*Elp1-201*	*Hmgb1-201*	*Ttbk2-202*
*Wnt7b-201*	*Elovl6-201*	*Chic1-201*	*Pck1-201*	*Zswim4-201*	*Aqp11-205*	*Mthfr-201*	*Gpr146-201*	*Mapk3-202*
*Lnx2-201*	*Psmc3-210*	*Slc38a3-201*	*Gtf2ird1-229*	*Ccdc39-201*	*Slc38a3-202*	*Oser1-201*	*P2ry1-203*	*Heatr1-206*
*Zfp386-204*	*Gprc5b-204*	*Serpind1-202*	*Fam47e-202*	*Arg1-201*	*Ankrd13c-202*	*Zfp592-201*	*Tmem98-201*	*Tmub2-202*
*Creg1-202*	*Calcoco1-201*	*Nme5-204*	*Map1lc3a-201*	*Mid1ip1-201*	*Ints6-201*	*Net1-201*	*Zfp322a-201*	*Rb1cc1-214*
*Rbm33-204*	*Pou2af2-202*	*Laptm4b-201*	*Dnajb11-203*	*Tbcel-203*	*Smad4-201*	*Ewsr1-205*	*Zkscan8-201*	*Kdm3a-201*
*Fam135a-206*	*Rpl30-201*	*Fads6-201*	*Ppp1r3g-201*	*Srsf1-205*	*Slc20a2-201*	*Slc9a3-203*	*Gpx6-201*	*Map4k4-209*
*Rnf38-202*	*Pde4b-207*	*Lrfn3-201*	*Dyrk1b-201*	*Ddx42-201*	*Map3k5-202*	*Hnrnpf-202*	*Cstf2t-201*	
*Ephx1-201*	*Sec24c-201*	*Gpam-202*	*Tacc2-205*	*Mbd5-203*	*Suds3-202*	*Crebbp-205*	*Tbc1d14-201*	
*Rtl5-201*	*Stard13-208*	*Raf1-201*	*Ttc38-203*	*Meiob-201*	*Plekhm1-201*	*Proca1-201*	*Acbd5-213*	
*Uqcc1-204*	*Mok-202*	*Aox1-201*	*Atat1-203*	*Fem1a-201*	*Net1-202*	*Rims2-201*	*Mtmr3-203*	
*Abcb4-201*	*Mrtfb-204*	*Ube2h-202*	*Wac-201*	*Cpeb2-204*	*Serpina3n-201*	*Shroom1-201*	*Cyth2-203*	
*Cpq-201*	*Ttll11-202*	*Zfp446-203*	*Fus-201*	*Csad-205*	*Lrp6-201*	*Ano1-203*	*Chrm3-202*	
*Heca-201*	*Septin9-204*	*Anks4b-201*	*Tomm40-202*	*Mul1-201*	*Fnbp1-210*	*Leng8-203*	*Opn3-201*	
*Nlgn3-201*	*Ctdsp2-202*	*Kctd7-201*	*Tesk1-201*	*Efr3a-212*	*Taok3-201*	*Pnn-201*	*H2az1-201*	
*Map3k13-203*	*Nfil3-201*	*Gbp7-201*	*Hlcs-201*	*Evi5-201*	*Mpv17l-201*	*Pon2-201*	*Ppp2r2d-201*	
*Btg1-201*	*Arl4a-201*	*Ypel2-201*	*Casp7-201*	*Ptpn21-203*	*Mettl1-201*	*Hmgcr-201*	*Nup50-201*	
*Tcp11l2-201*	*Zfp740-201*	*Plec-218*	*Rrp9-201*	*Khnyn-203*	*Wdr45-204*	*Mtss1-201*	*Magi1-203*	
*Tmem64-201*	*Pcdh1-203*	*Atosa-201*	*Hnrnpd-211*	*Cyp39a1-204*	*Kcna2-202*	*Ephb6-201*	*Mterf2-201*	
*Zfp13-201*	*Hsdl2-201*	*Txndc11-202*	*Ankrd46-203*	*Zdhhc2-201*	*Dtx4-201*	*Itgb1-201*	*Abhd8-201*	
*Azin1-201*	*Aldh1l1-203*	*Nhlrc1-201*	*Echdc3-201*	*Lrrfip2-205*	*Rbbp6-202*	*Tmx2-201*	*Akap8-206*	
*Emc2-201*	*Gpr17-201*	*Znrf3-201*	*Dnmbp-206*	*Lcorl-212*	*Rabl3-201*	*Acaca-201*	*Csad-201*	
*Tgoln1-201*	*Sppl3-201*	*Pomk-201*	*Triobp-203*	*Rnf11-201*	*Phf13-201*	*Otud3-201*	*Rdx-204*	
*Pde4dip-201*	*Tlcd4-203*	*Zfp715-203*	*Stat1-206*	*Axin1-201*	*Tmem44-204*	*Ranbp10-203*	*Snap25-201*	

**Table 2:** List of transcripts with significantly altered expression in brains of *Lrp5*^−/−^ mice compared to *Wt* mice. *p* value < 0.05.

Gene Transcripts with Altered Expression in Brains of *Lrp5*^−/−^ Mice				
*Fgfbp3-201*	*Pde4d-202*	*Cramp1-201*	*Ttyh1-201*	*Brap-205*
*Eps8l2-206*	*Ankrd33b-202*	*H2-Q7-201*	*Erich5-201*	*Ighg2c-202*
*Rab11fip3-201*	*Cask-210*	*Gm17167-201*	*Ube2d2a-210*	*Abi1-205*
*Lrp5-202*	*Zfp386-204*	*Gm8116-201*	*Bcat2-205*	*Atp6v1c1-202*
*Gm12191-201*	*Rpl30-201*	*Aldh1l1-204*	*Baalc-202*	*Ywhaz-203*
*Rbfox1-202*	*Ciz1-202*	*Atp6v1c1-201*	*Slc29a1-222*	*Lzts3-202*
*Ndn-201*	*Atg16l2-211*	*Fn1-204*	*Rpl30-ps9-201*	*Rspo2-201*
*Hax1-207*	*Gm8276-201*	*Cobl-210*	*Ankrd46-204*	*Pak3-210*
*Ptpn6-203*	*Marveld2-201*	*Btaf1-201*	*Gm54215-201*	*Meg3-201*
*Ankrd33b-203*	*Ywhaz-207*	*Eif3s6-ps2-201*	*Ywhaz-201*	

**Table 3:** Altered functions in the brains of *Lrp5*^−/−^ mice according to functional gene enrichment analysis. The 1st column indicates the altered function; the 2nd column shows the *p* value associated with each function; the 3rd column shows the Gene Ontology subhierarchy associated with the altered function; the 4th column lists the transcripts with altered expression in the brains of *Lrp5*^−/−^ mice that are associated with the altered function (GO:BP stands for Gene Ontology:Biological Process; GO:CC stands for Gene Ontology:Cellular Component; GO:MF stands for Gene Ontology:Molecular Function).

Altered Function	*p*-Value	Source	Significantly Altered Transcripts
Cell morphogenesis involved in differentiation	0.00713631	GO:BP	*Necdin-201; Ptpn6-203; Cask-210; Fn1-204; Cobl-210; Abi-205; Ltzs3-202; Pak3-210*
Cell morphogenesis involved in neuron differentiation	0.03526747	GO:BP	*Necdin-201; Cask-210; Fn1-204; Cobl-210; Abi-205; Ltzs3-202; Pak3-210*
Postsynaptic density	0.00032878	GO:CC	*Cask-210; Rpl30-201; Ywhaz-207; Baalc-202; Abi1-205; Ltzs3-202; Pak3-210*
Postsynapse	0.00037269	GO:CC	*Rab11fip3-201; Slc29a1-222; Cask-210; Rpl30-201; Ywhaz-207; Baalc-202; Abi1-205; Ltzs3-202; Pak3-210*
Asymmetric synapse	0.00043165	GO:CC	*Cask-210; Rpl30-201; Ywhaz-207; Baalc-202; Abi1-205; Ltzs3-202; Pak3-210*
Postsynaptic specialization	0.00060115	GO:CC	*Cask-210; Rpl30-201; Ywhaz-207; Baalc-202; Abi1-205; Ltzs3-202; Pak3-210*
Neuron to neuron synapse	0.00072925	GO:CC	*Cask-210; Rpl30-201; Ywhaz-207; Baalc-202; Abi1-205; Ltzs3-202; Pak3-210*
Cell junction	0.00111901	GO:CC	*Rab11fip3-201; Ptpn6-203; Cask-210; Rpl30-201; Ywhaz-207; Baalc-202; Slc29a1-222; Marveld2-201; Atp6v1c1-201; Ttyh1-201; Abi1-205; Ltzs3-202; Pak3-210*
Synapse	0.00187814	GO:CC	*Rab11fip3-201; Slc29a1-222; Cask-210; Rpl30-201; Ywhaz-207; Baalc-202; Atp6v1c1-201; Abi1-205; Ltzs3-202; Pak3-210*
Apical part of cell	0.01692298	GO:CC	*Hax1-207; Pde4d-202; Marveld2-201; Atp6v1c1-201; Fn1-204; Cobl-210*
Plasma membrane region	0.01918133	GO:CC	*Rab11fip3-201; Eps8l2-206; Hax1-207; Pde4d-202; Cask-210; Marveld2-201; Fn1-204; Ttyh1-201; Slc29a1-222*
Protein domain specific binding	0.00720761	GO:MF	*Hax1-207; Ptpn6-203; Cask-210; Ywhaz-207; Fn1-204; Abi1-205; Lzts3-202; Pak3-210*
Protein binding	0.04096533	GO:MF	*Fgfbp3-201; Eps8l2-206; Rab11fip3-201; Ndn-201; Hax1-207; Ptpn6-203; Pde4d-204; Cask-210; Marveld2-201; Ywhaz-207; Fn1-204; Cobl-210; Ankrd46-204; Abi1-205; Lzts3-202; Pak3-210; Lrp5-202; Ankrd33b-206; Ciz1-202; Atg16l2-211; H2-Q7-201; Aldh1l1-204; Btaf1-201; Ube2d2a-210; Brap-205; Ighg2c-202; Rspo2-201*

**Table 4:** Altered functions in livers of *Lrp5*^−/−^ mice according to functional gene enrichment analysis. The 1st column indicates the altered process; the 2nd column shows the *p* value associated with each function; the 3rd column shows the Gene Ontology subhierarchy associated with the altered function; the 4th column shows the number of altered transcripts associated with the function. Only the 28 functions with the smallest *p* values are listed, as more than 300 functions were altered in the livers of *Lrp5*^−/−^ mice (based on the Gene Ontology database) (GO:BP stands for Gene Ontology:Biological Process; GO:CC stands for Gene Ontology:Cellular Component; GO:MF stands for Gene Ontology:Molecular Function).

Altered Function	*p*-Value	Source	Number of Significantly Altered Transcripts
Regulation of cellular metabolic process	1.54 × 10^−19^	GO:BP	193
Regulation of cellular process	2.68 × 10^−18^	GO:BP	325
Regulation of primary metabolic process	4.22 × 10^−18^	GO:BP	199
Biological regulation	1.31 × 10^−16^	GO:BP	347
Reguation of metabolic process	2.41 × 10^−16^	GO:BP	223
Regulation of nitrogen compound metabolic process	7.82 × 10^−16^	GO:BP	189
Regulation of biological process	1.66 × 10^−15^	GO:BP	337
Organic substance biosynthetic process	3.49 × 10^−15^	GO:BP	190
Biosynthetic process	3.75 × 10^−15^	GO:BP	192
Cellular process	6.86 × 10^−15^	GO:BP	446
Positive regulation of biological process	1.45 × 10^−14^	GO:BP	207
Positive regulation of cellular process	4.05 × 10^−14^	GO:BP	190
Regulation of macromolecule metabolic process	2.91 × 10^−13^	GO:BP	204
Cellular metabolic process	2.50 × 10^−12^	GO:BP	299
Cellular biosynthetic process	3.84 × 10^−12^	GO:BP	173
Regulation of biosynthetic process	1.43 × 10^−11^	GO:BP	143
Organonitrogen compund metabolic process	3.46 × 10^−11^	GO:BP	192
Anatomical structural development	4.95 × 10^−11^	GO:BP	186
Developmental process	6.58 × 10^−11^	GO:BP	199
Metabolic process	1.18 × 10^−10^	GO:BP	344
Primary metabolic process	1.37 × 10^−10^	GO:BP	318
Negative regulation of cellular process	1.92 × 10^−10^	GO:BP	157
Regulation of macromolecule biosynthetic process	3.05 × 10^−10^	GO:BP	133
Regulation of cellular biosynthetic process	3.16 × 10^−10^	GO:BP	136
Multicellular organism development	3.37 × 10^−10^	GO:BP	154
Positive regulation of cellular metabolic process	6.09 × 10^−10^	GO:BP	111
System development	6.42 × 10^−10^	GO:BP	136
Localization	1.11 × 10^−9^	GO:BP	166
	·		
	·		
	·		

## Data Availability

The data that support the findings of this study are available from the corresponding author (MBP) upon reasonable request.

## Supplementary Materials

The supporting information can be downloaded at https://www.mdpi.com/1422-0067/25/12/6763/s1.
