# Supplementary material for: Unique Splicing of Lrp5 in the Brain: A New Player in Neurodevelopment and Brain Maturation
Source: Int J Mol Sci. 2024 Jun 20;25(12):6763. doi: 10.3390/ijms25126763 (PMC11203723; doi:10.3390/ijms25126763)
Supplement: Supplementary file 1 [file ijms-25-06763-s001.zip › ijms-3038429-supplementary.pdf]

Supplementary Figure S1

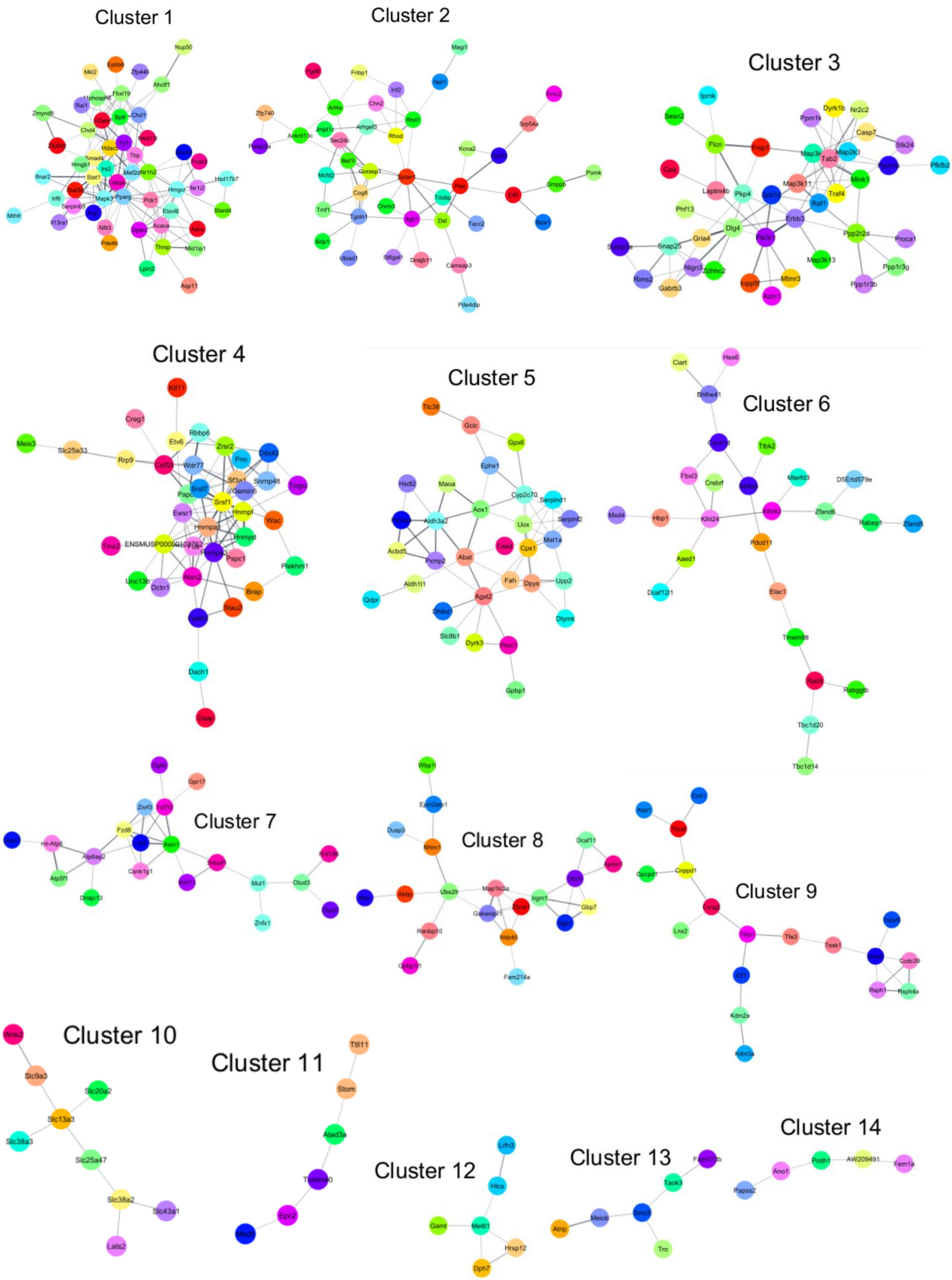

Supplementary Table S1

Cluster #	Enrichment retrieval	Term name	FDR value
Cluster 1	Regulation of cellular biosynthetic process	GO:0031326	4.33×10^{-9}
	Regulation of transcription	GO:0006355	3.82×10^{-8}
Cluster 2	Transport to the Golgi and subsequent modification	MMU-948021	8.48×10^{-5}
	Organelle	GO:0043226	0.005
Cluster 3	Positive regulation of signalling	GO:0023056	6.11×10^{-10}
	Regulation of phosphate metabolic processes	GO:0019220	3.56×10^{-9}
Cluster 4	Nucleic acid binding	GO:0003676	8.53×10^{-14}
	mRNA splicing	MMU-72172	6.48×10^{-13}
Cluster 5	Small molecule metabolic process	GO:0044281	6.75×10^{-10}
	Cellular catabolic process	GO:0044248	3.12×10^{-8}
Cluster 6	Circadian rhythm	CL:4564	2.5×10^{-4}
Cluster 7	WNT Signalling Pathway	KW-0879	7.88×10^{-6}
Cluster 8	Autophagy	GO:0006914	4.1×10^{-4}
Cluster 9	Mixed, incl. axoneme assembly, and Trichohyalin-plectin-homology domain	CL:11382	0.0017
Cluster 10	Transport of inorganic cations/anions and amino acids/oligopeptides	MMU-425393	2.83×10^{-7}
Cluster 11	Ganglion	BTO:0000497	0.0372
Cluster 13	DNA repair	GO:0006281	0.029

Supplementary Figure S1. Clustering of liver protein-protein interaction network. Clustering was performed using Glay clustering algorithm in Cytoscape software. Clustering resulted in the formation of 14 isolated clusters. Data available upon demand to authors.

Supplementary Table S1. Functional gene enrichment analysis was performed separately for each of the clusters generated using Glay clustering algorithm. Table shows cellular functions altered in Lrp5-/- mice livers, with the corresponding term name and a FDR value calculated by STRING enrichment algorithm in Cytoscape software.
